# Crystal structure of K_0.75_[Fe^II^_3.75_Fe^III^_1.25_(HPO_3_)_6_]·0.5H_2_O, an open-framework iron phosphite with mixed-valent Fe^II^/Fe^III^ ions

**DOI:** 10.1107/S2056989015024007

**Published:** 2016-01-01

**Authors:** Edurne S. Larrea, José Luis Mesa, Estibaliz Legarra, Andrés Tomás Aguayo, Maria Isabel Arriortua

**Affiliations:** a Dpto. Mineralogía y Petrología, Universidad del País Vasco, UPV/EHU, Sarrina s/n, 48940 Leioa, Spain; b Dpto. Química Inorgánica, Universidad del País Vasco, UPV/EHU, Sarrina s/n, 48940 Leioa, Spain; c Basque Center for Materials, Applications & Nanostructures (BCMaterials), Parque Tecnológico de Zamudio, Camino de Ibaizabal, Edificio 500-1°, 48160 Derio, Spain; d Dpto. Ingeniería Química, Universidad del País Vasco, UPV/EHU, Sarrina s/n, 48940 Leioa, Spain

**Keywords:** crystal structure, open-framework structure, hydro­thermal synthesis, mixed-valent Fe^II^/Fe^III^ compound, isotypism

## Abstract

Single crystals of the title compound, potassium hexa­phosphito­penta­ferrate(II,III) hemihydrate, K_0.75_[Fe^II^_3.75_Fe^III^_1.25_(HPO_3_)_6_]·0.5H_2_O, were grown under mild hydro­thermal conditions. The crystal structure is isotypic with Li_1.43_[Fe^II^_4.43_Fe^III^_0.57_(HPO_3_)_6_]·1.5H_2_O and (NH_4_)_2_[Fe^II^_5_(HPO_3_)_6_] and exhibits a [Fe^II^_3.75_Fe^III^_1.25_(HPO_3_)_6_]^0.75−^ open framework with disordered K^+^ (occupancy 3/4) as counter-cations. The anionic framework is based on (001) sheets of two [FeO_6_] octa­hedra (one with point group symmetry 3.. and one with point group symmetry .2.) linked along [001] through [HPO_3_]^2−^ oxoanions. Each sheet is constructed from 12-membered rings of edge-sharing [FeO_6_] octa­hedra, giving rise to channels with a radius of *ca* 3.1 Å where the K^+^ cations and likewise disordered water mol­ecules (occupancy 1/4) are located. O⋯O contacts between the water mol­ecule and framework O atoms of 2.864 (5) Å indicate hydrogen-bonding inter­actions of medium strength. The infrared spectrum of the compound shows vibrational bands typical for phosphite and water groups. The Mössbauer spectrum is in accordance with the presence of Fe^II^ and Fe^III^ ions.

## Abstract 2

K_0.7_[Fe^II^_3.7_Fe^III^_1.3_(HPO_3_)_6_]·5H_2_O was synthesized under mild hydro­thermal conditions. The open-framework phosphite contains channels extending along [001] in which disordered potassium cations and water mol­ecules are located.

## Chemical context   

Open-framework materials have been a major research topic in materials science during the last decades because of their potential applications (Barrer, 1982 ▸; Wilson *et al.*, 1982 ▸; Davis, 2002 ▸, Adams & Pendlebury, 2011 ▸). Many efforts have been made to obtain porous materials using different oxoanions in combination with metals (Yu & Xu, 2010 ▸). The use of structure-directing agents or templates, not only organic but also inorganic, has also been extended in order to achieve this purpose. In this context, a new porous mixed-valent Fe^II^/Fe^III^ phosphitoferrate with lithium cations and an open-framework structure, Li_1.43_[Fe^II^_4.43_Fe^III^_0.57_(HPO_3_)_6_]·1.5H_2_O, has been reported (Chung *et al.*, 2011 ▸). This structure presents channels of *ca* 5.5 Å diameter along the [001] direction in which water mol­ecules and lithium ions are located. The same type of framework but with Fe^II^ cations and with ammonium counter-anions was reported recently for (NH_4_)_2_[Fe^II^_5_(HPO_3_)_6_] (Berrocal *et al.*, 2014 ▸).

Here we report on the synthesis and crystal structure of isotypic K_0.75_[Fe^II^_3.75_Fe^III^_1.25_(HPO_3_)_6_]·0.5H_2_O resulting from the replacement of lithium/ammonium by potassium. The iron cations in this compound are again in a mixed valence oxidation state of +II and +III.

## Structural commentary   

**Figure 1:**
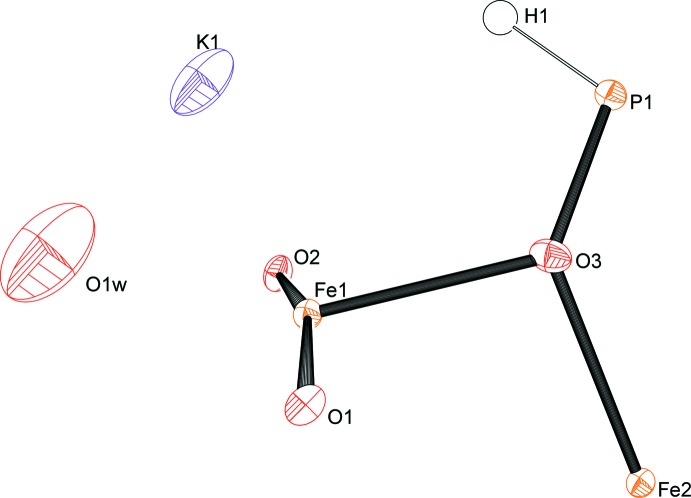
Asymmetric unit of K_0.75_[Fe^II^_3.75_Fe^III^_1.25_(HPO_3_)_6_]·0.5H_2_O with displacement parameters drawn at the 50% probability level.

**Figure 2:**
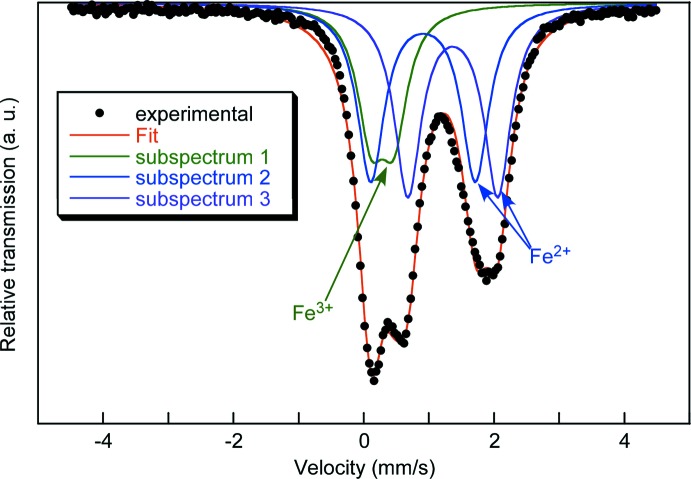
Mössbauer spectrum of the title compound showing the presence of Fe^II^ and Fe^III^. The fit was made with the *NORMOS* program (Brand *et al.*, 1983 ▸).

The asymmetric unit of K_0.75_[Fe^II^_3.75_Fe^III^_1.25_(HPO_3_)_6_]·0.5H_2_O (Fig. 1 ▸) contains two Fe sites on special positions (6*f* and 4*d*) with site symmetries of .2. and .3., respectively, three O sites, one P site and one H site. In addition, disordered sites associated with a water mol­ecule (O1*W*) and the potassium counter-cation are present. The crystal structure is made up of two types of [FeO_6_] octa­hedra linked *via* edge-sharing into sheets parallel to (001). These sheets consist of 12-membered rings formed by six [Fe1O_6_] octa­hedra and six [Fe2O_6_] octa­hedra. In one of the FeO_6_ octa­hedra (Fe1), the Fe—O bond lengths range from 2.046 (2) to 2.179 (2) Å while in the [Fe2O_6_] octa­hedron, a more uniform bond-length distribution from 2.134 (2) to 2.143 (2) is observed. In order to assign the content of Fe^II^ and Fe^III^ on these sites, a Mössbauer spectrum was recorded (Fig. 2 ▸). Three different components were observed, two doublets, corresponding to Fe^II^ cations, and a third doublet, corresponding to Fe^III^ cations. The determined Fe^II^/Fe^III^ ratio is 3.1, in good agreement with the formula. According to bond-valence calculations (Brown, 2002 ▸), a clear assignment of which of the two iron sites carries the Fe^III^ cations cannot be made. The calculated bond-valence sum for site Fe1 assuming Fe^II^ is 2.213 valence units (v.u.), while assuming Fe^III^ gives 2.367. Corresponding values for the Fe2 site are 2.014 v.u. assuming Fe^II^ and 2.155 assuming Fe^III^. The O—Fe—O bond angles of the two [FeO_6_] octa­hedra are in the range between 78.10 (8) and 102.63 (7)° for *cis*- and between 175.77 (11) and 163.23 (8)° for the *trans*-angles.

**Figure 3:**
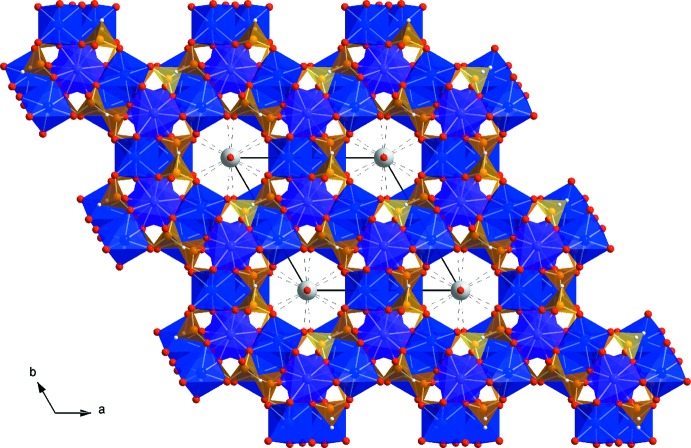
Crystal structure of K_0.75_[Fe^II^_3.75_Fe^III^_1.25_(HPO_3_)_6_]·0.5H_2_O in polyhedral representation, in a projection along [001]. Colour code: Fe1O_6_ octa­hedra are blue, Fe2O_6_ octa­hedra are magenta, HPO_3_ tetra­hedra are orange, O atoms are red and K^+^ ions are grey. Hydrogen-bonding inter­actions between O1 from the framework and O1*W* are shown with dashed lines.

The (001) iron oxide sheets are linked through phosphite groups whereby six anions share the innermost oxygen atoms of each ring (Fig. 3 ▸), forming 12-membered channels extending along [001]. The channels have a radius of about 3.1 Å. The P—O bond lengths of the anion range from 1.529 (2) to 1.541 (2) Å and are comparable with those of the two isotypic structures. The P—H distance in the title compound is 1.29 (4) Å, and the O—P—O bond angles range from 110.24 (11) to 114.32 (11)°.

The disordered potassium cations and water mol­ecules are located on special positions in the twelve-membered channels of the framework with site symmetries of 32. and 3.., respectively. The occupancy factors are 0.75 for potassium and 0.25 for the water mol­ecule. Although the hydrogen atoms of the water mol­ecule could not be located, the O⋯O distance of 2.864 (5) Å between the water O1*W* atom and the O1 atom of the framework indicates possible hydrogen-bonding inter­actions of medium strength. Because the O1*W* site is located on a threefold rotation axis, three hydrogen bonds with the inorganic skeleton with an angle of 113.42 (5)° are possible.

## Synthesis and characterization   

K_0.75_[Fe^II^_3.75_Fe^III^_1.25_(HPO_3_)_6_]·0.5H_2_O was synthesized under mild hydro­thermal conditions and autogeneous pressure (10–20 bar at 343 K). The reaction mixture was prepared from 30 ml water, 2 ml of hypo­phospho­rous acid, 0.17 mmol of KOH and 0.37 mmol of FeCl_3·_·6H_2_O. The mixture had a pH value of ≃ 3.0. The reaction mixture was sealed in a polytetra­fluoro­ethyl­ene (PTFE)-lined steel pressure vessel, which was maintained at 343 K for five days. This procedure allowed the formation of single crystals of the title compound with a dark green to black colour.

The IR spectrum (see supporting information for this submission) shows typical bands corresponding to the stretching and deformation mode of the water mol­ecules at 3235 and 2410 cm^−1^, respectively. The spectrum also shows the stretching and deformation modes of the P—H bond at 1750 cm^−1^. The bands corresponding to the symmetric (ν_s_) and anti­symmetric (ν_as_) stretching vibrational modes of the (PO_3_) groups appear at 930 and 1151 cm^−1^, whereas the symmetric (δ_s_) and anti­symmetric (δ_as_) deformation modes of this group are centred at 450 and 590 cm^−1^ (Nakamoto, 1997 ▸; Chung *et al.*, 2011 ▸).

Thermogravimetric analysis of the title compound (see supporting information for this submission) shows a first mass-loss process of 1.05% between room temperature and 498 K. This mass loss corresponds to the removal of water (theoretical value: 1.13%). Between 498 K and 673 K, another mass loss of 0.45% takes place which could not be assigned to a chemical reaction. This second process is followed by a third continuous process associated with a considerable gain of mass due to the oxidation of the compound.

## Refinement   

Crystal data, data collection and structure refinement details are summarized in Table 1 ▸. H atoms of the water mol­ecule were not modelled. The hydrogen atom of the phosphite group was located in a difference density map and was refined without any constraint. Potassium and water oxygen sites are located in the channels. The occupancy factors of both atoms were initially set taking into account the previous characterization (themogravimetric measurement, Mössbauer spectrum fit). Some trials to refine the occupancy factors of these atoms were made. However, the results were very similar to those initially set, with a slight increase of reliability factors. Therefore, for the final model the occupancy factors were fixed at 0.75 for the K1 and at 0.25 for the O1*W* site.

## Supplementary Material

Crystal structure: contains datablock(s) I, 4R. DOI: 10.1107/S2056989015024007/wm5216sup1.cif

Structure factors: contains datablock(s) I. DOI: 10.1107/S2056989015024007/wm5216Isup2.hkl

Supporting information file. DOI: 10.1107/S2056989015024007/wm5216Isup4.tif

Supporting information file. DOI: 10.1107/S2056989015024007/wm5216Isup5.tif

CCDC reference: 1442401

Additional supporting information:  crystallographic information; 3D view; checkCIF report

## Figures and Tables

**Table 1:** Experimental details

Crystal data	
Chemical formula	K_0.75_[Fe^II^_3.75_Fe^III^_1.25_(HPO_3_)_6_]·0.5H_2_O
*M*_r_	797.45
Crystal system, space group	Trigonal, *P* 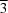 *c*1
Temperature (K)	100
*a*, *c* (Å)	10.1567 (5), 9.2774 (6)
*V* (Å^3^)	828.82 (8)
*Z*	2
Radiation type	Mo *K*α
μ (mm^−1^)	5.14
Crystal size (mm)	0.29 × 0.05 × 0.04

Data collection	
Diffractometer	Agilent SuperNova
Absorption correction	Analytical (*CrysAlis PRO*; Agilent, 2014 ▸)
*T*_min_, *T*_max_	0.423, 0.845
No. of measured, independent and observed [*I* > 2σ(*I*)] reflections	5132, 647, 618
*R*_int_	0.026
(sin θ/λ)_max_ (Å^−1^)	0.664

Refinement	
*R*[*F* ^2^ > 2σ(*F* ^2^)], *wR*(*F* ^2^), *S*	0.026, 0.061, 1.19
No. of reflections	647
No. of parameters	54
H-atom treatment	All H-atom parameters refined
Δρ_max_, Δρ_min_ (e Å^−3^)	0.8, −0.50

Computer programs: *CrysAlis PRO* (Agilent, 2014 ▸), *OLEX2* (Dolomanov, 2009 ▸), *SHELXL2014* (Sheldrick, 2015 ▸), *DIAMOND* (Brandenburg, 2001 ▸) and *WinGX* (Farrugia, 2012 ▸).

## supplementary crystallographic information

### Crystal data

**Table:**

K_0.75_[FeII3.75FeIII_1.25_(HPO_3_)_6_]·0.5H_2_O	*D*_x_ = 3.195 Mg m^−^^3^
*M**_r_* = 797.45	Mo *K*α radiation, λ = 0.71073 Å
Trigonal, *P*3*c*1	Cell parameters from 3002 reflections
Hall symbol: -P 3 2"c	θ = 2.3–28.0°
*a* = 10.1567 (5) Å	µ = 5.14 mm^−^^1^
*c* = 9.2774 (6) Å	*T* = 100 K
*V* = 828.82 (8) Å^3^	Prism, black
*Z* = 2	0.29 × 0.05 × 0.04 mm
*F*(000) = 779	

### Data collection

**Table:**

Agilent SuperNova diffractometer	647 independent reflections
Radiation source: Nova (Mo) X-ray micro-source	618 reflections with *I* > 2σ(*I*)
Multilayer optics monochromator	*R*_int_ = 0.026
Detector resolution: 16.2439 pixels mm^-1^	θ_max_ = 28.2°, θ_min_ = 2.3°
ω scans	*h* = −13→12
Absorption correction: analytical (*CrysAlis PRO*; Agilent, 2014)	*k* = −9→13
*T*_min_ = 0.423, *T*_max_ = 0.845	*l* = −12→10
5132 measured reflections	

### Refinement

**Table:**

Refinement on *F*^2^	Primary atom site location: iterative
Least-squares matrix: full	Secondary atom site location: difference Fourier map
*R*[*F*^2^ > 2σ(*F*^2^)] = 0.026	Hydrogen site location: difference Fourier map
*wR*(*F*^2^) = 0.061	All H-atom parameters refined
*S* = 1.19	*w* = 1/[σ^2^(*F*_o_^2^) + (0.0254*P*)^2^ + 2.2546*P*] where *P* = (*F*_o_^2^ + 2*F*_c_^2^)/3
647 reflections	(Δ/σ)_max_ = 0.015
54 parameters	Δρ_max_ = 0.8 e Å^−^^3^
0 restraints	Δρ_min_ = −0.50 e Å^−^^3^

### Special details

**Table:**

Geometry. All s.u.'s (except the s.u. in the dihedral angle between two l.s. planes) are estimated using the full covariance matrix. The cell s.u.'s are taken into account individually in the estimation of s.u.'s in distances, angles and torsion angles; correlations between s.u.'s in cell parameters are only used when they are defined by crystal symmetry. An approximate (isotropic) treatment of cell s.u.'s is used for estimating s.u.'s involving l.s. planes.
Refinement. Refinement of *F*^2^ against ALL reflections. The weighted *R*-factor *wR* and goodness of fit *S* are based on *F*^2^, conventional *R*-factors *R* are based on *F*, with *F* set to zero for negative *F*^2^. The threshold expression of *F*^2^ > 2σ(*F*^2^) is used only for calculating *R*-factors(gt) *etc*. and is not relevant to the choice of reflections for refinement. *R*-factors based on *F*^2^ are statistically about twice as large as those based on *F*, and *R*- factors based on ALL data will be even larger.

### Fractional atomic coordinates and isotropic or equivalent isotropic displacement parameters (Å^2^)

**Table:**

	*x*	*y*	*z*	*U*_iso_*/*U*_eq_	Occ. (<1)
Fe1	0.62108 (5)	0	0.25	0.00709 (16)	
Fe2	0.6667	0.3333	0.33159 (7)	0.00659 (18)	
P1	0.70307 (8)	0.11196 (8)	0.58780 (7)	0.00748 (18)	
O3	0.6916 (2)	0.1569 (2)	0.4316 (2)	0.0110 (4)	
O2	0.3954 (2)	−0.1473 (2)	0.3120 (2)	0.0095 (4)	
O1	0.8210 (2)	0.1342 (2)	0.1437 (2)	0.0124 (4)	
K1	1	0	0.25	0.0247 (5)	0.75
O1W	1	0	0.063 (2)	0.033 (4)	0.25
H1	0.645 (4)	−0.033 (4)	0.592 (4)	0.011 (9)*	

### Atomic displacement parameters (Å^2^)

**Table:**

	*U*^11^	*U*^22^	*U*^33^	*U*^12^	*U*^13^	*U*^23^
Fe1	0.0070 (2)	0.0067 (3)	0.0075 (3)	0.00336 (14)	−0.00016 (10)	−0.00031 (19)
Fe2	0.0062 (2)	0.0062 (2)	0.0074 (3)	0.00310 (11)	0	0
P1	0.0072 (3)	0.0087 (3)	0.0068 (3)	0.0042 (3)	0.0006 (2)	0.0003 (2)
O3	0.0112 (10)	0.0147 (10)	0.0083 (9)	0.0075 (8)	0.0018 (7)	0.0024 (8)
O2	0.0091 (9)	0.0079 (9)	0.0113 (9)	0.0041 (8)	0.0023 (7)	−0.0004 (7)
O1	0.0153 (10)	0.0095 (10)	0.0140 (10)	0.0073 (9)	−0.0003 (8)	0.0002 (8)
K1	0.0151 (7)	0.0151 (7)	0.0439 (15)	0.0075 (3)	0	0
O1W	0.018 (5)	0.018 (5)	0.062 (12)	0.009 (2)	0	0

### Geometric parameters (Å, º)

**Table:**

Fe1—O1	2.046 (2)	P1—H1	1.29 (4)
Fe1—O1^i^	2.046 (2)	O2—P1^vii^	1.534 (2)
Fe1—O2	2.096 (2)	O2—Fe2^viii^	2.134 (2)
Fe1—O2^i^	2.096 (2)	O1—P1^ix^	1.529 (2)
Fe1—O3^i^	2.179 (2)	O1—K1	2.935 (2)
Fe1—O3	2.179 (2)	K1—O1^x^	2.935 (2)
Fe1—K1	3.8486 (6)	K1—O1^xi^	2.935 (2)
Fe2—O2^i^	2.134 (2)	K1—O1^xii^	2.935 (2)
Fe2—O2^ii^	2.134 (2)	K1—O1^xiii^	2.935 (2)
Fe2—O2^iii^	2.134 (2)	K1—O1^i^	2.935 (2)
Fe2—O3	2.143 (2)	K1—Fe1^xii^	3.8486 (6)
Fe2—O3^iv^	2.143 (2)	K1—Fe1^x^	3.8486 (6)
Fe2—O3^v^	2.143 (2)	K1—K1^xiv^	4.6387 (3)
P1—O1^vi^	1.529 (2)	K1—K1^xv^	4.6387 (3)
P1—O2^vii^	1.534 (2)	O1W—O1W^xv^	1.17 (4)
P1—O3	1.541 (2)		
			
O1—Fe1—O1^i^	97.50 (12)	O1^xi^—K1—O1^xii^	63.23 (8)
O1—Fe1—O2	167.07 (8)	O1^x^—K1—O1^xiii^	63.23 (8)
O1^i^—Fe1—O2	89.81 (8)	O1^xi^—K1—O1^xiii^	109.30 (4)
O1—Fe1—O2^i^	89.81 (8)	O1^xii^—K1—O1^xiii^	78.54 (8)
O1^i^—Fe1—O2^i^	167.07 (8)	O1^x^—K1—O1	109.30 (4)
O2—Fe1—O2^i^	85.10 (11)	O1^xi^—K1—O1	78.54 (8)
O1—Fe1—O3^i^	90.97 (8)	O1^xii^—K1—O1	109.30 (4)
O1^i^—Fe1—O3^i^	91.82 (8)	O1^xiii^—K1—O1	171.12 (8)
O2—Fe1—O3^i^	78.11 (8)	O1^x^—K1—O1^i^	78.54 (8)
O2^i^—Fe1—O3^i^	98.73 (7)	O1^xi^—K1—O1^i^	109.30 (4)
O1—Fe1—O3	91.82 (8)	O1^xii^—K1—O1^i^	171.12 (8)
O1^i^—Fe1—O3	90.97 (8)	O1^xiii^—K1—O1^i^	109.30 (4)
O2—Fe1—O3	98.73 (7)	O1—K1—O1^i^	63.23 (8)
O2^i^—Fe1—O3	78.11 (8)	O1^x^—K1—Fe1^xii^	140.73 (4)
O3^i^—Fe1—O3	175.77 (11)	O1^xi^—K1—Fe1^xii^	31.62 (4)
O1—Fe1—K1	48.75 (6)	O1^xii^—K1—Fe1^xii^	31.62 (4)
O1^i^—Fe1—K1	48.75 (6)	O1^xiii^—K1—Fe1^xii^	94.44 (4)
O2—Fe1—K1	137.45 (6)	O1—K1—Fe1^xii^	94.44 (4)
O2^i^—Fe1—K1	137.45 (6)	O1^i^—K1—Fe1^xii^	140.73 (4)
O3^i^—Fe1—K1	92.11 (5)	O1^x^—K1—Fe1^x^	31.62 (4)
O3—Fe1—K1	92.11 (5)	O1^xi^—K1—Fe1^x^	140.73 (4)
O2^i^—Fe2—O2^ii^	85.13 (8)	O1^xii^—K1—Fe1^x^	94.44 (4)
O2^i^—Fe2—O2^iii^	85.13 (8)	O1^xiii^—K1—Fe1^x^	31.62 (4)
O2^ii^—Fe2—O2^iii^	85.13 (8)	O1—K1—Fe1^x^	140.73 (4)
O2^i^—Fe2—O3	78.10 (8)	O1^i^—K1—Fe1^x^	94.44 (4)
O2^ii^—Fe2—O3	93.44 (7)	Fe1^xii^—K1—Fe1^x^	120
O2^iii^—Fe2—O3	163.22 (8)	O1^x^—K1—Fe1	94.44 (4)
O2^i^—Fe2—O3^iv^	163.23 (8)	O1^xi^—K1—Fe1	94.44 (4)
O2^ii^—Fe2—O3^iv^	78.10 (8)	O1^xii^—K1—Fe1	140.73 (4)
O2^iii^—Fe2—O3^iv^	93.44 (7)	O1^xiii^—K1—Fe1	140.73 (4)
O3—Fe2—O3^iv^	102.63 (7)	O1—K1—Fe1	31.62 (4)
O2^i^—Fe2—O3^v^	93.44 (7)	O1^i^—K1—Fe1	31.62 (4)
O2^ii^—Fe2—O3^v^	163.22 (8)	Fe1^xii^—K1—Fe1	120
O2^iii^—Fe2—O3^v^	78.10 (8)	Fe1^x^—K1—Fe1	120
O3—Fe2—O3^v^	102.63 (7)	O1^x^—K1—K1^xiv^	109.64 (4)
O3^iv^—Fe2—O3^v^	102.63 (7)	O1^xi^—K1—K1^xiv^	70.36 (4)
O1^vi^—P1—O2^vii^	112.13 (11)	O1^xii^—K1—K1^xiv^	109.64 (4)
O1^vi^—P1—O3	114.32 (11)	O1^xiii^—K1—K1^xiv^	70.36 (4)
O2^vii^—P1—O3	110.24 (11)	O1—K1—K1^xiv^	109.64 (4)
O1^vi^—P1—H1	105.9 (16)	O1^i^—K1—K1^xiv^	70.36 (4)
O2^vii^—P1—H1	105.6 (16)	Fe1^xii^—K1—K1^xiv^	90
O3—P1—H1	108.1 (16)	Fe1^x^—K1—K1^xiv^	90
P1—O3—Fe2	135.47 (12)	Fe1—K1—K1^xiv^	90
P1—O3—Fe1	123.82 (12)	O1^x^—K1—K1^xv^	70.36 (4)
Fe2—O3—Fe1	98.26 (8)	O1^xi^—K1—K1^xv^	109.64 (4)
P1^vii^—O2—Fe1	127.45 (12)	O1^xii^—K1—K1^xv^	70.36 (4)
P1^vii^—O2—Fe2^viii^	130.31 (12)	O1^xiii^—K1—K1^xv^	109.64 (4)
Fe1—O2—Fe2^viii^	101.19 (8)	O1—K1—K1^xv^	70.36 (4)
P1^ix^—O1—Fe1	129.84 (13)	O1^i^—K1—K1^xv^	109.64 (4)
P1^ix^—O1—K1	124.86 (11)	Fe1^xii^—K1—K1^xv^	90
Fe1—O1—K1	99.63 (7)	Fe1^x^—K1—K1^xv^	90
O1^x^—K1—O1^xi^	171.12 (8)	Fe1—K1—K1^xv^	90
O1^x^—K1—O1^xii^	109.30 (4)	K1^xiv^—K1—K1^xv^	180

Symmetry codes: (i) *x*−*y*, −*y*, −*z*+1/2; (ii) *y*+1, *x*, −*z*+1/2; (iii) −*x*+1, −*x*+*y*+1, −*z*+1/2; (iv) −*y*+1, *x*−*y*, *z*; (v) −*x*+*y*+1, −*x*+1, *z*; (vi) −*y*+1, −*x*+1, *z*+1/2; (vii) −*x*+1, −*y*, −*z*+1; (viii) *y*, *x*−1, −*z*+1/2; (ix) −*y*+1, −*x*+1, *z*−1/2; (x) −*y*+1, *x*−*y*−1, *z*; (xi) −*x*+2, −*x*+*y*+1, −*z*+1/2; (xii) −*x*+*y*+2, −*x*+1, *z*; (xiii) *y*+1, *x*−1, −*z*+1/2; (xiv) −*x*+2, −*y*, −*z*+1; (xv) −*x*+2, −*y*, −*z*.
