# Supplementary figures and images for: Crystal structure of K0.75[FeII 3.75FeIII 1.25(HPO3)6]·0.5H2O, an open-framework iron phosphite with mixed-valent FeII/FeIII ions
Source: Acta Crystallogr E Crystallogr Commun. 2016 Jan 1;72(Pt 1):63–5. doi: 10.1107/S2056989015024007 (PMC4704752; doi:10.1107/S2056989015024007)

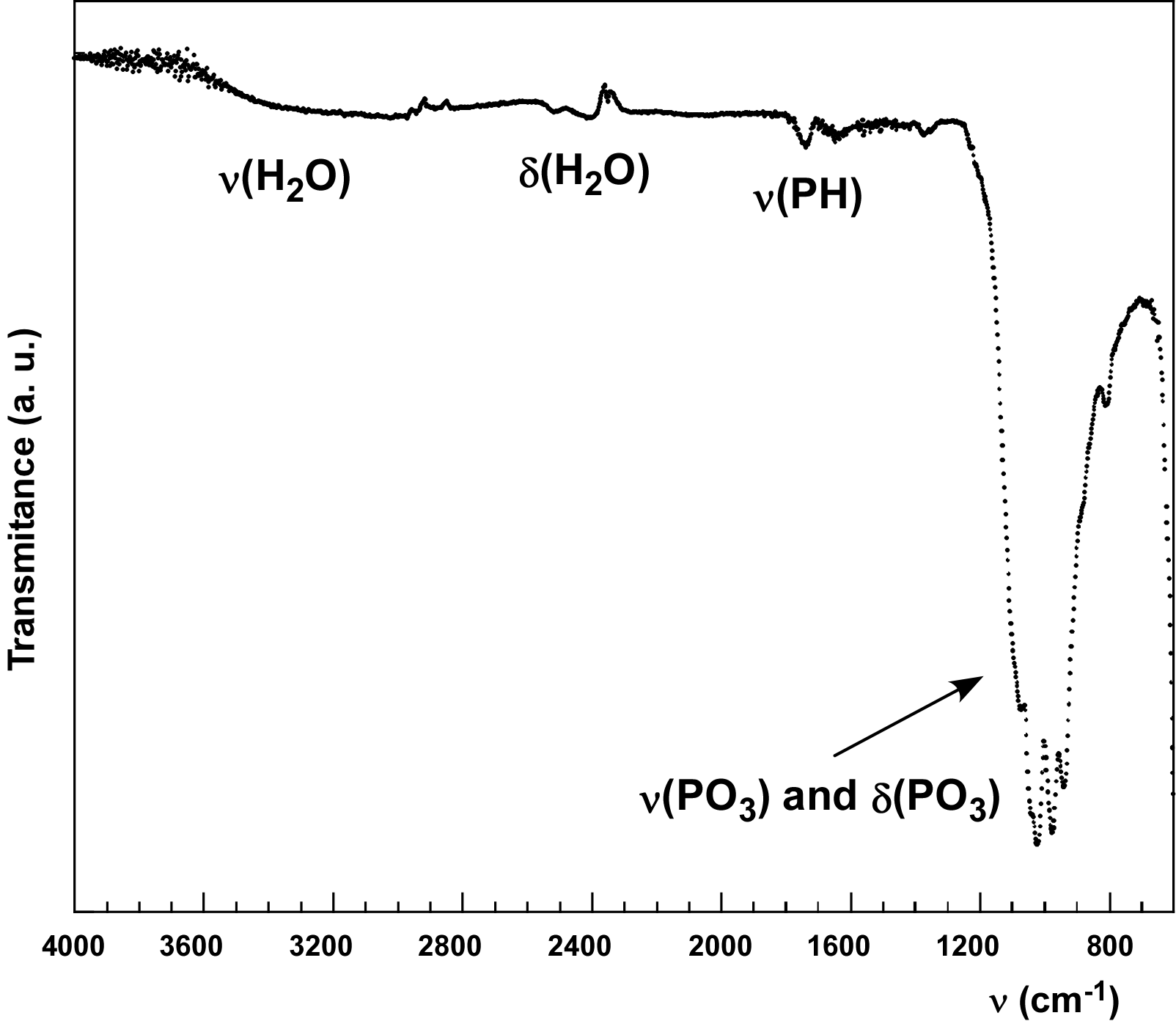

Supplement: Supplementary file 3 [file e-72-00063-Isup4.tif]

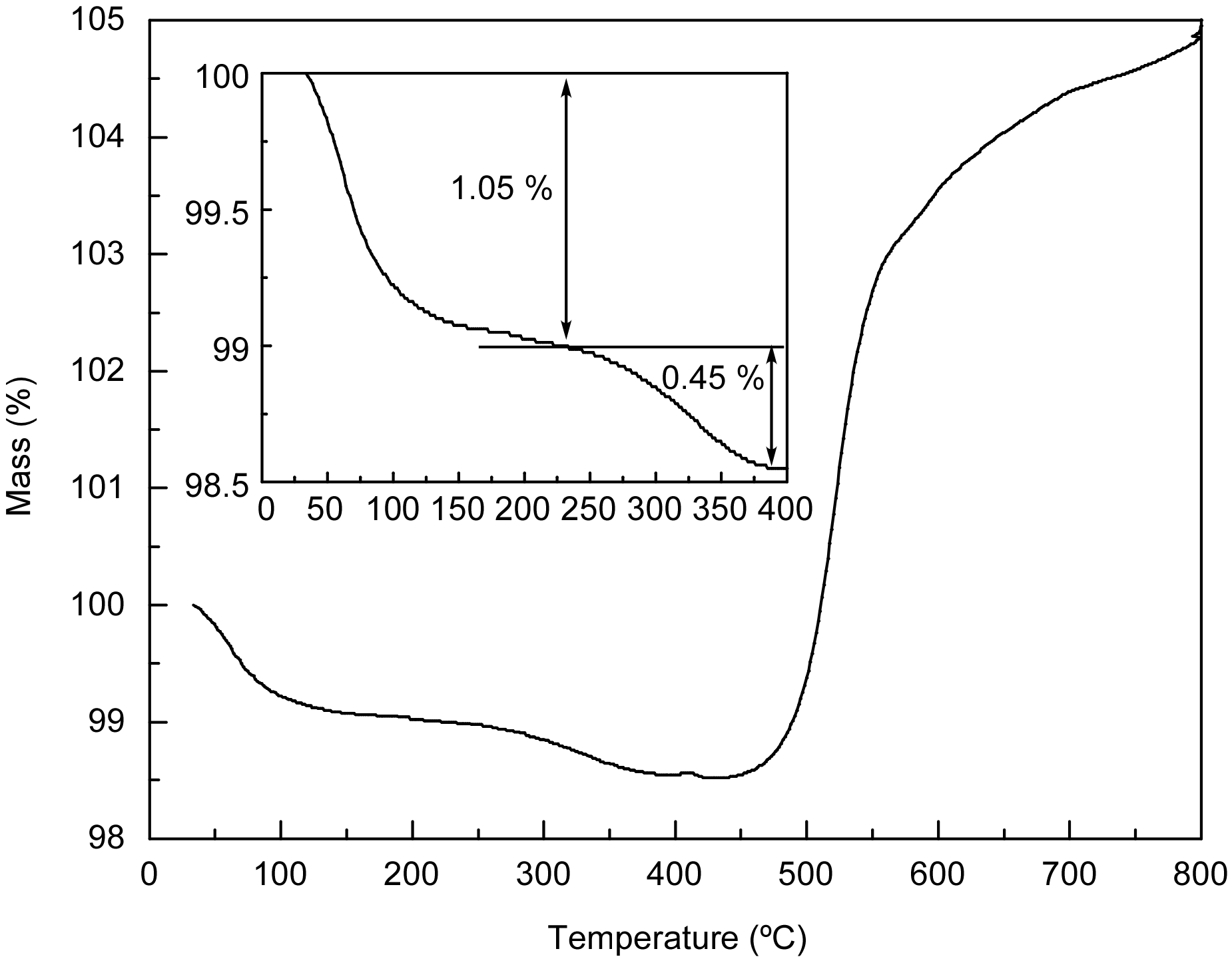

Supplement: Supplementary file 4 [file e-72-00063-Isup5.tif]
